# A multifunctional bilayer wound dressing co-loaded with nanosilver and bFGF for enhanced skin regeneration: synergistic antibacterial, hemostatic, and angiogenic effects

**DOI:** 10.3389/fbioe.2025.1650545

**Published:** 2025-10-17

**Authors:** Qianqian Wang, Fang Ma, Ying Xian, Xingyan Shi, Shenglan Ma, Rui Zhang, Hualin Zhang

**Affiliations:** ^1^ Department of Prosthodontics, College of Stomatology, Ningxia Medical University, Yinchuan, China; ^2^ Department of Stomatology, The People’s Hospital of Wenxi, Wenxi, China; ^3^ Ningxia Province Key Laboratory of Oral Diseases Research, Ningxia Medical University, Yinchuan, China; ^4^ Department of General Stomatology, General Hospital of Ningxia Medical University, Yinchuan, China

**Keywords:** bilayer dressing, nanosilver, bFGF, electrospun membrane, chitosan sponge

## Abstract

**Introduction:**

Wound closure, infection prevention and accelerated healing are important to consider in the development of multifunctional dressings. In this study, we aimed to design a dressing that can simulate the structure of normal skin, prevent bacterial infection, improve hemostasis and angiogenesis, accelerate cell adhesion and proliferation, and continuously promote skin regeneration.

**Methods:**

We created a multifunctional bilayer dressing coloaded with nanosilver and basic fibroblast growth factor (bFGF) via emulsion electrospinning and vacuum freeze-drying methods. The upper hydrophobic layer was composed of a poly(lactic‐co‐glycolic acid)/wool keratin (PLGA/WK) electrospun membrane containing nanosilver and bFGF, and the lower hydrophilic layer was composed of a chitosan sponge containing bFGF.

**Results and Discussion:**

The bilayer dressing allowed the sustained release of nanosilver and bFGF for more than 2 weeks, had good water absorption and water retention rates, had an appropriate water vapor transmission rate, absorbed excess exudate, and maintained a moist wound environment. *In vitro*, the dressing prevented bacterial penetration, accelerated fibroblast migration, and promoted angiogenesis and coagulation. Furthermore, the bilayer dressing facilitated cell migration and proliferation, promoted vessel formation, shortened the healing time and significantly promoted wound healing *in vivo*. By day 3, the wound healing rate in the bilayer dressing group (35.28% ± 2.06%) was significantly higher than that in the control group (23.99% ± 4.32%), representing an increase of approximately 47%. Our findings suggest that this multifunctional bilayer dressing coloaded with nanosilver and bFGF is a potential candidate for skin repair and regeneration applications.

## 1 Introduction

The skin is the body’s first defensive barrier against external environments (Kong et al., 2019). When the skin is traumatized, covering the surface of the wound with a dressing can provide a suitable healing environment. Ideal wound dressings should have the following attributes to promote wound healing (Lin et al., 2024; Liang et al., 2021): good water absorption and moisturizing properties; good biocompatibility; no toxicity; no induction of inflammation or allergic or other adverse reactions; appropriate mechanical properties; effective anti-inflammatory and antibacterial properties; good biodegradability; and a proper drug loading capacity with sustained drug release.

To date, traditional wound dressings are still the most commonly used method for treating skin wounds, but they act only as temporary barriers and do not have antibacterial effects or actively induce tissue regeneration and repair (Liu et al., 2022). Therefore, we aimed to construct a dressing coloaded with an antimicrobial agent and a growth factor with sustained drug release properties. The antibiotic can be continuously released to the wound surface to reduce the incidence of wound infection and the toxic side effects of systemic drugs. Moreover, the continuous release of the growth factor can actively regulate cell functions, thus accelerating wound healing.

Small silver particles less than 100 nm in size are referred to as nanosilver. Compared with large metallic silver, nanosilver has a larger specific surface area and more significant antibacterial effects (Liu et al., 2024). It can sterilize a variety of gram-positive and gram-negative bacteria, including multidrug-resistant bacteria, and the application of nanosilver rarely results in drug resistance (Liu et al., 2018). However, owing to the thermodynamic instability of nanosilver, agglomeration easily occurs, greatly reducing its antibacterial properties.

bFGF is an important mitogen during wound repair. bFGF affects cell growth, proliferation, migration and differentiation during wound healing and can promote the wound healing process (Tu et al., 2025). However, owing to the short half-life of growth factors in the body and their degradation by proteases activated around the wound, the biological activities of bFGF in the body cannot be maintained for a long period. Therefore, if nanosilver and bFGF can be encapsulated in a sustained-release material, the agglomeration of the nanosilver can be prevented, bFGF activity can be protected, and their action durations can be prolonged, thus improving their therapeutic effects.

Owing to the complexity of different types of wounds and the wound healing process, most single-layer dressings are not effective when applied to all types of wounds and during all stages of wounds because of their limited characteristics, nor can they simulate normal skin structure (Zandi et al., 2021). Bilayer dressings are usually composed of a thin, dense upper layer and a loose, porous lower layer, which simulate the epidermal and dermal structures of the skin well. Moreover, different functional drugs can be added to each layer so that various aspects of wound treatment can be met during different healing periods (Mirhaj et al., 2022).

Therefore, in this study, a multifunctional bilayer dressing containing an antibacterial drug and a growth factor was constructed. The upper layer was composed of a PLGA/WK emulsion–electrospun fibrous membrane containing nanosilver and bFGF. The nanosilver was encapsulated in the shell of the fibers, while bFGF was encapsulated in the core, allowing the sustained release of these drugs to occur during different stages of wound healing. The lower layer was a porous chitosan sponge containing bFGF. Furthermore, the physicochemical properties, drug release *in vitro*, antibacterial performance, cytocompatibility, angiogenic and blood coagulation effects *in vitro*, and ability to repair full-thickness skin wounds *in vivo* of the bilayer dressing were evaluated. The dual drug-loaded multifunctional bilayer dressing developed in this study may have potential applications in skin wound repair.

## 2 Materials and methods

### 2.1 Materials

PLGA (Mw: 5 × 10^4^ g/mol) was purchased from Jufukai Biotechnology Co., Ltd. (Jinan, China). Wool keratin was obtained from Shouhe Biotechnology Co., Ltd. (Xi’an, China). Dextran (DEX) and bovine serum albumin (BSA) were purchased from Sigma–Aldrich (United States). Nanosilver-loaded calcium alginate microsphere powders were prepared by our research team via the chemical crosslinking of calcium ions. Chitosan (deacetylation degree ≥95%) was purchased from Aladdin (Shanghai, China). bFGF was purchased from PeproTech (United States). *Staphylococcus aureus* (*S. aureus,* ATCC6538) and *Escherichia coli* (*E. coli*, ATCC25922) were obtained from the Beijing Biological Preservation Center.

### 2.2 Animals

Thirty-six healthy Sprague–Dawley (SD) rats (6–8 weeks old, male, SPF grade) were provided by the Experimental Animal Department of Ningxia Medical University, China. All animal experiments complied with the ARRIVE guidelines and were performed in accordance with the United Kingdom Animals (Scientific Procedures) Act, 1986, and the associated guidelines. The experiments were approved by the Animal Ethics Committee of Ningxia Medical University.

### 2.3 Preparation and characterization of PLGA/WK electrospun membranes containing nanosilver and bFGF

#### 2.3.1 Preparation of the electrospinning solution

DEX was thoroughly dissolved in PBS, and then the prepared bFGF/BSA solution was added to the DEX solution to achieve a final bFGF concentration of 1 g/L and a final DEX concentration of 15% w/v (Zhang et al., 2020). The prepared DEX/bFGF/BSA solution was labeled the aqueous solution. Additionally, PLGA was dissolved in a mixture of trichloromethane and N,N-dimethylformamide (DMF) to prepare a 15% w/v PLGA solution to which 1.0% w/v wool keratin powder was added. Afterward, 0%, 0.2%, 0.5%, and 1.0% w/v nanosilver-loaded calcium alginate microsphere powders were thoroughly mixed to generate PLGA/WK solutions with different concentrations of nanosilver, which were labeled the oil phase solutions. Afterward, the aqueous solution was added to each oil phase solution dropwise at a water-to-oil volume ratio of 1:13 with stirring at 4 °C for 30 min to obtain four types of PLGA/WK electrospun solutions containing bFGF (0.07 g/L) and different concentrations of nanosilver.

#### 2.3.2 Preparation of the PLGA/WK electrospun membranes containing nanosilver and bFGF

Four types of PLGA/WK electrospun membranes containing bFGF and different amounts of nanosilver were obtained from four electrospinning solutions via emulsion electrospinning using a previously reported method (Zhang et al., 2022). Finally, the four types of membranes contained 0%, 0.2%, 0.5% and 1.0% nanosilver and were named the 0%, 0.2%, 0.5%, and 1.0% groups, respectively.

#### 2.3.3 Morphological observations

The surface morphologies of the four types of electrospun membranes were observed by scanning electron microscopy (SEM; JSM-6510, JEOL, Japan), and their fiber diameters were measured using Nano Measurer software. Additionally, the four types of electrospun fibers were collected on copper mesh and examined by transmission electron microscopy (TEM; JEM-3010, JEOL, Japan) to determine whether they had stable core–shell structures.

#### 2.3.4 X-ray diffraction analysis

The crystal structures of the electrospun membranes were analyzed by X-ray diffraction (D8 ADVANCE, Bruker, Germany) at an accelerating voltage of 40 kV and a current of 40 mA and scanned at 5°/min from 5 to 90°.

#### 2.3.5 Fourier transform infrared (FTIR) spectroscopy

The FTIR spectra of the electrospun membranes were acquired with a Fourier transform infrared spectrometer with an attenuated total reflection apparatus (Tensor 27, Bruker, Germany) in the range of 4,000 cm^-1^ to 400 cm^-1^.

#### 2.3.6 Amount of drug encapsulated and the drug encapsulation rate

The four types of electrospun membranes (n = 4) were weighed and added to a digestion tube containing 10 mL of a mixture of nitric acid and perchloric acid. After the samples had completely dissolved, the digestion tube was placed into a preheated instrument for acid digestion. When the bottom of the tube contained a crystalline salt, the tube was removed, the crystallized salt was dissolved, and the solution was diluted and subjected to further testing.

The content of nanosilver encapsulated in the membrane samples was determined with an inductively coupled plasma emission spectrometer (710-ES, Varian, United States). The encapsulation rate of nanosilver was subsequently calculated using the following formula:

$$\text{Encapsulation rate}\left(\%\right)=\left(\frac{\text{Amount}\;\text{of}\;\text{nanosilver}\;\text{in}\;\text{the}\;\text{membrane}}{\text{Amount}\;\text{of}\;\text{nanosilver}\;\text{added}}\right)\times 100\%.$$

#### 2.3.7 *In Vitro* analysis of nanosilver release

The four types of electrospun membranes (n = 4) were weighed and placed in a centrifuge tube with 10 mL of PBS and then oscillated at a rate of 100 r/min at 37 °C. After 1 h, 2 h, 4 h, 8 h, 12 h, 24 h, 4 days, 7 days, 10 days and 14 days (14 days in total), the liquid in the centrifuge tube was removed as the test solution, and a new 10 mL of fresh PBS was added. After the test solution was subjected to microwave digestion, the silver ion content was determined using an inductively coupled plasma optical emission spectrometer.

#### 2.3.8 *In Vitro* analysis of antibacterial activity

The four types of electrospun membranes (n = 4) were cut, sterilized, and placed on nutrient agar plates coated with an *S. aureus* or *E. coli* solution. Afterward, the plates were incubated at 37 °C for 14 days. Photographs were taken after 1, 4, 7, 10 and 14 days of incubation, at which time the diameter of the inhibition zone was measured.

#### 2.3.9 Cytotoxicity

Disks of the four types of membranes (diameter: 1.5 cm; n = 4) were cocultured with L-929 cells at a density of 2.5 × 10^4^ cells/well in 24-well plates. CCK-8 assays were subsequently performed after 1, 3, 5 and 7 days of incubation. The cell survival rate was calculated using the following formula, and the cytotoxicity of the membranes was evaluated according to the ISO 10993–5 standard (Loloei et al., 2024):

$$\text{Cell survival rate}\left(\%\right)=\left(\frac{\text{OD}\;\text{value}\;\text{in}\;\text{the}\;\text{experimental}\;\text{group}}{\text{OD}\;\text{value}\;\text{in}\;\text{the}\;\text{control}\;\text{group}}\right)\times 100\%$$

#### 2.3.10 Cell adhesion

The four types of sterilized membranes (n = 4) were placed at the bottom of 24-well plates. L-929 cells were subsequently inoculated on the membranes at a density of 1 × 10^5^ cells/mL and cultured in a CO_2_ incubator. After 3 days, the adhesion of the cells to the electrospun membranes was observed by SEM.

### 2.4 Preparation and characterization of the chitosan sponges containing bFGF

#### 2.4.1 Preparation of the chitosan sponges containing bFGF

Chitosan sponges were prepared by vacuum freeze-drying according to our previously reported method (Ma et al., 2024). The chitosan sponges were then soaked in 95% ethanol for 2 h and then in a 10% sodium hydroxide solution for another 2 h. After being cleaned with ultrapure water, the washed chitosan sponges were placed in a 24-well plate, prefrozen, and immediately transferred to a vacuum freeze dryer (LGJ-12 A, Sihuanqihang, China) for 12 h. Afterward, the sponges were soaked in 500 μL of solutions containing 0, 500, 1,000 or 1,500 ng of bFGF at 4 °C for 2 h, after which the sponges were freeze-dried for 12 h in a vacuum freeze dryer. Finally, four types of chitosan sponges containing 0, 500, 1,000 and 1,500 ng of bFGF were obtained and named the 0 ng, 500 ng, 1,000 ng and 1,500 ng groups, respectively.

#### 2.4.2 Morphological observations

The four types of dried sponge samples were attached to copper stubs using a conductive adhesive. After the samples were sputtered with gold for 55 s, their surface and cross-sectional morphologies were observed by SEM. The pore size and porosity of the samples were measured with an automatic mercury porosimeter (Auto Pore IV 9500, Micromeritics, United States).

#### 2.4.3 *In Vitro* release of bFGF

Samples of the chitosan sponges containing bFGF (n = 4) were placed in centrifuge tubes filled with 4 mL of PBS and shaken at 37 °C and 100 r/min. At predetermined time points (1 h, 4 h, 8 h, 24 h, 3 days, 7 days and 14 days), 1 mL of solution was removed for storage at −20 °C. Additionally, 1 mL of fresh PBS solution was added to each tube after sampling. The amount of bFGF in each sample was measured with an ELISA kit, and these data were used to construct a release curve. The cumulative release rate of bFGF was calculated via the following formula:

$$\text{Cumulative release rate}\left(\%\right)=\frac{\text{bFGF}\;\text{content}\;\text{in}\;\text{sample}\;\text{solution}}{\text{total}\;\text{bFGF}\;\text{content}}\times 100\%$$

#### 2.4.4 Cytotoxicity

The cytotoxicity evaluation was performed according to the procedure described in Section 2.3.9.

#### 2.4.5 Cell adhesion

The cell adhesion evaluation was performed according to the procedure described in Section 2.3.10.

### 2.5 Preparation and characterization of the multifunctional bilayer dressing loaded with nanosilver and bFGF

#### 2.5.1 Preparation of the multifunctional bilayer dressing loaded with nanosilver and bFGF

The PLGA/WK solution containing bFGF and the optimal content of nanosilver prepared in Section 2.3 was used as the electrospinning solution to construct the dressing, and the chitosan sponge containing the optimal content of bFGF prepared in Section 2.4 was fixed on the roller of the electrospinning apparatus to collect the nanofibers. A bilayer dressing coloaded with nanosilver and bFGF was subsequently prepared by emulsion electrospinning. The upper layer was composed of a PLGA/WK electrospun membrane containing 0.2% nanosilver and bFGF, and the lower layer was composed of a chitosan sponge containing 500 ng of bFGF.

#### 2.5.2 Morphological observations

The cross-sectional morphology of the bilayer dressing was observed via SEM.

#### 2.5.3 Water absorption rate, water retention rate and water vapor transmission rate (WVTR)

The water absorption rate, water retention rate and WVTR of the three types of materials (the bilayer dressing, the PLGA/WK electrospun membrane containing 0.2% nanosilver and bFGF, and the chitosan sponge containing 500 ng of bFGF; n = 4) were determined using previously reported methods (Ma et al., 2024). In brief, the PLGA/WK electrospun membrane containing 0.2% nanosilver and bFGF and the chitosan sponge containing 500 ng of bFGF were considered the upper layer and the lower layer, respectively.

#### 2.5.4 Water contact angle measurements

The contact angles of the upper and lower layers of the bilayer dressing were measured with a contact angle measuring instrument (JY-82B, DataPhysics, Germany).

#### 2.5.5 Bacterial penetration test

Circular samples of the sterile gauze, upper layer, lower layer, and bilayer dressing with diameters of 15 mm were prepared, whereas the control group did not include any sample material (n = 3). Additionally, *S. aureus* and *E. coli* suspensions were diluted to a concentration of 1 × 10^9^ colony forming units (CFUs)/mL. Each of the above materials was sterilized and placed on an agar plate onto which 20 µL of bacterial suspension was added and evenly coated. After an incubation at 37 °C for 24 h, the agar blocks underlying each sample were removed and transferred to a centrifuge tube containing 2 mL of PBS, and the surfaces were washed repeatedly with PBS to detach the bacteria. The samples in the centrifuge tubes were diluted, and 100 µL of each diluted solution was routinely coated on new agar plates and cultured at 37 °C for 24 h. The number of bacterial colonies that formed on each plate was recorded, from which the amount of bacteria contained in the original PBS solution was calculated.

#### 2.5.6 Cell scratch wound healing assays

Several evenly spaced, parallel horizontal lines were drawn on the back of the 6-well plate, with at least 5 lines per well. L-929 cells were then inoculated into 6-well plates at a density of 5 × 10^5^ cells per well. When the cell confluence reached 100%, a sterilized pipette tip (200 μL) was used to make a scratch in the cell layer perpendicular to the horizontal lines, and then the cells were washed 3 times with PBS to remove the detached cells and cell debris. Next, samples of the upper layer, lower layer, bilayer dressing (n = 3) and 2 mL of serum-free medium were added to each well, and the plates were cultured in an incubator and photographed at 0, 6, 12 and 24 h. The images of each group were analyzed with ImageJ software, and the cell migration rate was calculated using the following formula:

$$\text{Cell migration rate}\left(\%\right)=\frac{\text{scratch}\;\text{area}\;\text{at}\;0\;h-\text{scratch}\;\text{area}\;\text{at}\;a\;\text{particular}\;\text{time}\;\text{point}}{\text{scratch}\;\text{area}\;\text{at}\;0\;h}\times 100\%$$

#### 2.5.7 Angiogenesis assessment

The upper layer, lower layer, and bilayer dressing samples (n = 3) were placed into a 12-well plate, and 1 mL of complete medium was added to each well. Afterward, the plate was incubated at 37 °C for 24 h to prepare the leaching solutions. Matrix glue was spread onto a 96-well plate, and HUVECs were seeded at a density of 3 × 10^4^ cells/well. Then, 100 μL of each type of leaching solution was added to the appropriate wells, and the samples were cultured at 37 °C. After 12 h, blood vessel formation was observed under an inverted optical microscope, and the cells were photographed. The total number of junctions in each group was determined with ImageJ software.

#### 2.5.8 Coagulation test

The upper layer, lower layer, and bilayer dressing samples (n = 3) were placed on a Petri dish, and 50 μL of SD rat whole blood was dropped onto the surface of each sample and incubated at 37 °C for 5 min. Then, 20 mL of deionized water was slowly poured from the edge of the Petri dish so that the red blood cells not embedded in blood clots would undergo hemolysis in the water. After 20 min, the absorbance value of each sample was measured at 540 nm with a microplate reader, and the coagulation index (BCI) was calculated using the following formula:

$$\text{BCI}=\left(\frac{\text{OD}\;\text{value}{\;}_{\text{experimental}\;\text{group}}}{\text{OD}\;\text{value}{\;}_{\text{control}\;\text{group}}}\right)\times 100$$

### 2.6 Full-thickness skin wound repair

#### 2.6.1 Construction of the full-thickness skin defect model

The following six groups were included in this test (Table 1): group a, blank control; group b, bilayer dressing without drugs; group c, bilayer dressing loaded with nanosilver and bFGF in the upper layer; group d, bilayer dressing loaded with bFGF in both the upper layer and lower layer; group e, bilayer dressing loaded with bFGF in the upper layer; and group f, bilayer dressing loaded with nanosilver and bFGF (n = 3). A full-thickness skin defect with a diameter of 1 cm was prepared on the backs of the SD rats, disinfected bilayer dressings were applied to the wounds, and medical gauze was applied on top.

**TABLE 1:** Details of the six groups analyzed in the animal experiment.

Groups	a	b	c	d	e	f
	Blank control	Bilayer dressing without drugs	Bilayer dressing loaded with nanosilver and bFGF in the upper layer	Bilayer dressing loaded with bFGF in both the upper and lower layers	Bilayer dressing loaded with bFGF in the upper layer	Bilayer dressing loaded with nanosilver and bFGF
Upper layer		PLGA/WK membrane	PLGA/WK membrane loaded with nanosilver and bFGF	PLGA/WK membrane loaded with bFGF	PLGA/WK membrane loaded with bFGF	PLGA/WK membrane coloaded with nanosilver and bFGF
Lower layer		Chitosan sponge	Chitosan sponge	Chitosan sponge loaded with bFGF	Chitosan sponge	Chitosan sponge loaded with bFGF

#### 2.6.2 Gross observations and evaluation of the wound healing rate

The skin wounds on the rats were observed after 3, 7, 14 and 21 days, and photographs were captured with a digital SLR camera. ImageJ software was used to analyze the wound healing rate, which was calculated using the following formula:

$$\text{Wound healing rate}\left(\%\right)=\left[\frac{\text{original}\;\text{wound}\;\text{area}-\text{wound}\;\text{area}\;\text{at}\;\text{each}\;\text{time}\;\text{point}}{\text{original}\;\text{wound}\;\text{area}}\right]\times 100\%$$

#### 2.6.3 2.6.3. Histological examination

At 3, 7, 14 and 21 days after wounding, the selected rats were anesthetized, and a 2–3 mm area of the wound edge on the rats’ back was removed. The samples were subjected to hematoxylin–eosin (HE) staining to observe the degree of inflammation in the new tissues and the formation of new blood vessels, epithelium and skin appendages. The expression of proliferating cell nuclear antigen (PCNA) in each group was evaluated by immunohistochemical staining, and the ratio of the positively stained area was calculated using the following formula:

$$\text{Positively stained area ratio}=\left(\frac{\text{positively}\;\text{stained}\;\text{area}}{\text{tissue}\;\text{area}}\right)\times 100\%$$

#### 2.6.4 Western blot

At 7 days after wounding, proteins were extracted from tissue samples from each group using a whole protein extraction kit and quantified with a protein quantification kit (Servicebio, China). The proteins from each sample were subsequently used to determine PCNA and CD34 expression via Western blot. The ratio of the intensity of the target band to the intensity of the internal reference band was used to determine the relative expression levels of the corresponding proteins.

### 2.7 Statistical analysis

SPSS 25.0 software was used for statistical analysis of the data, and all the data are presented as the means ± standard deviations ( $\bar{X}\pm S$ ). Multiple comparisons were performed using one-way analysis of variance (ANOVA) and Tukey’s *post hoc* test, and *p* < 0.05 was considered to indicate statistical significance.

## 3 Results

### 3.1 Characterization of the PLGA/WK electrospun membranes containing nanosilver and bFGF

#### 3.1.1 Morphological observations

**FIGURE 1:**
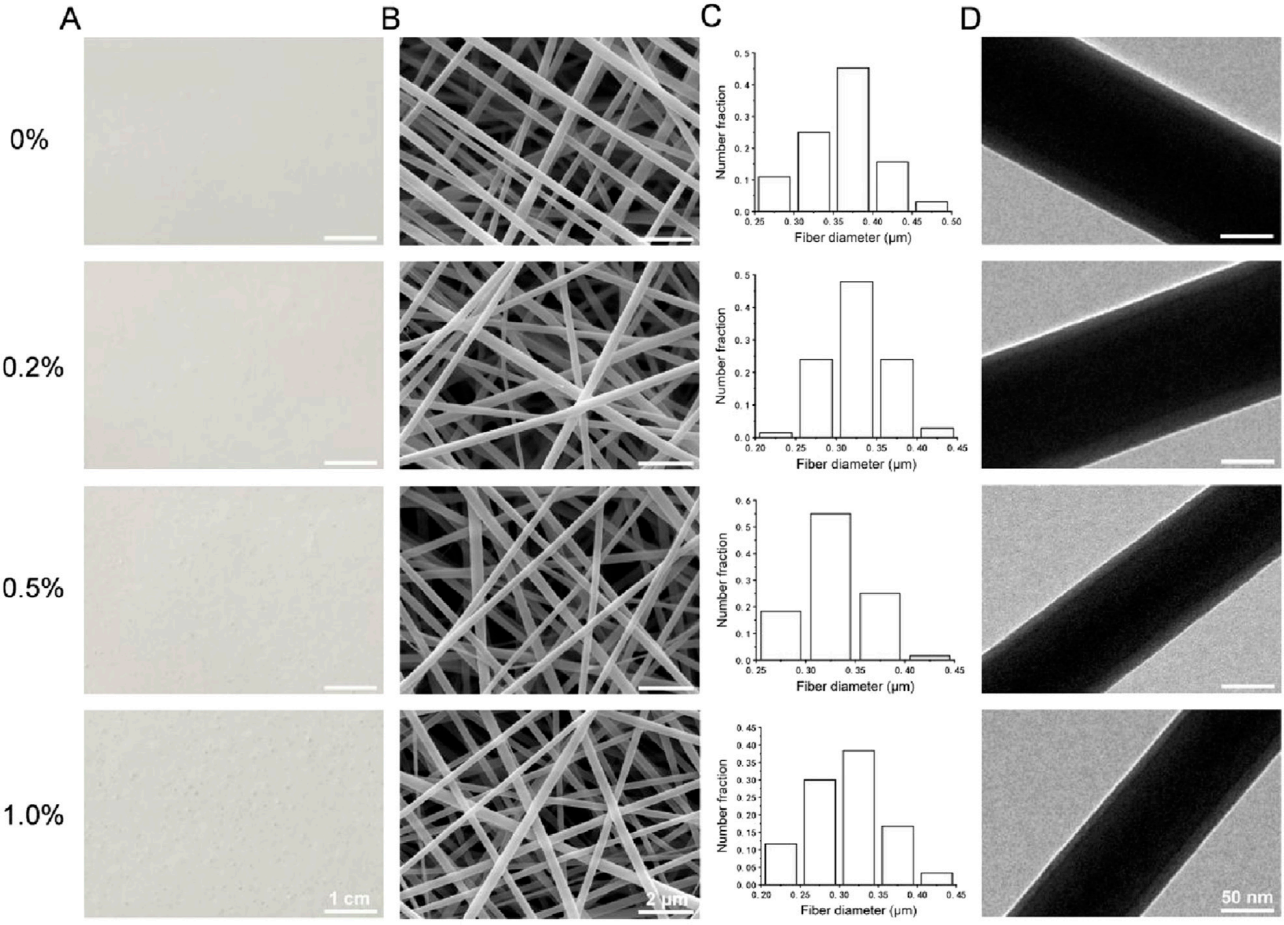
**(A)** Photographs of the four types of membranes. **(B)** SEM images. **(C)** Diameter distributions. **(D)** TEM images.

Photographs and SEM images of the four types of membranes are shown in Figures 1A,B. The four types of membranes were composed of fibers with smooth surfaces and no obvious beaded structures. The diameter distributions of the fibers in each group, which were all on the nanoscale, are shown in Figure 1C. The average fiber diameters of the 0%, 0.2%, 0.5%, and 1.0% groups were 0.36 ± 0.05 μm, 0.32 ± 0.04 μm, 0.32 ± 0.04 μm, and 0.31 ± 0.05 μm, respectively. TEM images of each type of fiber are shown in Figure 1D. All of the fibers had a stable core–shell structure.

#### 3.1.2 X-ray diffraction analysis

**FIGURE 2:**
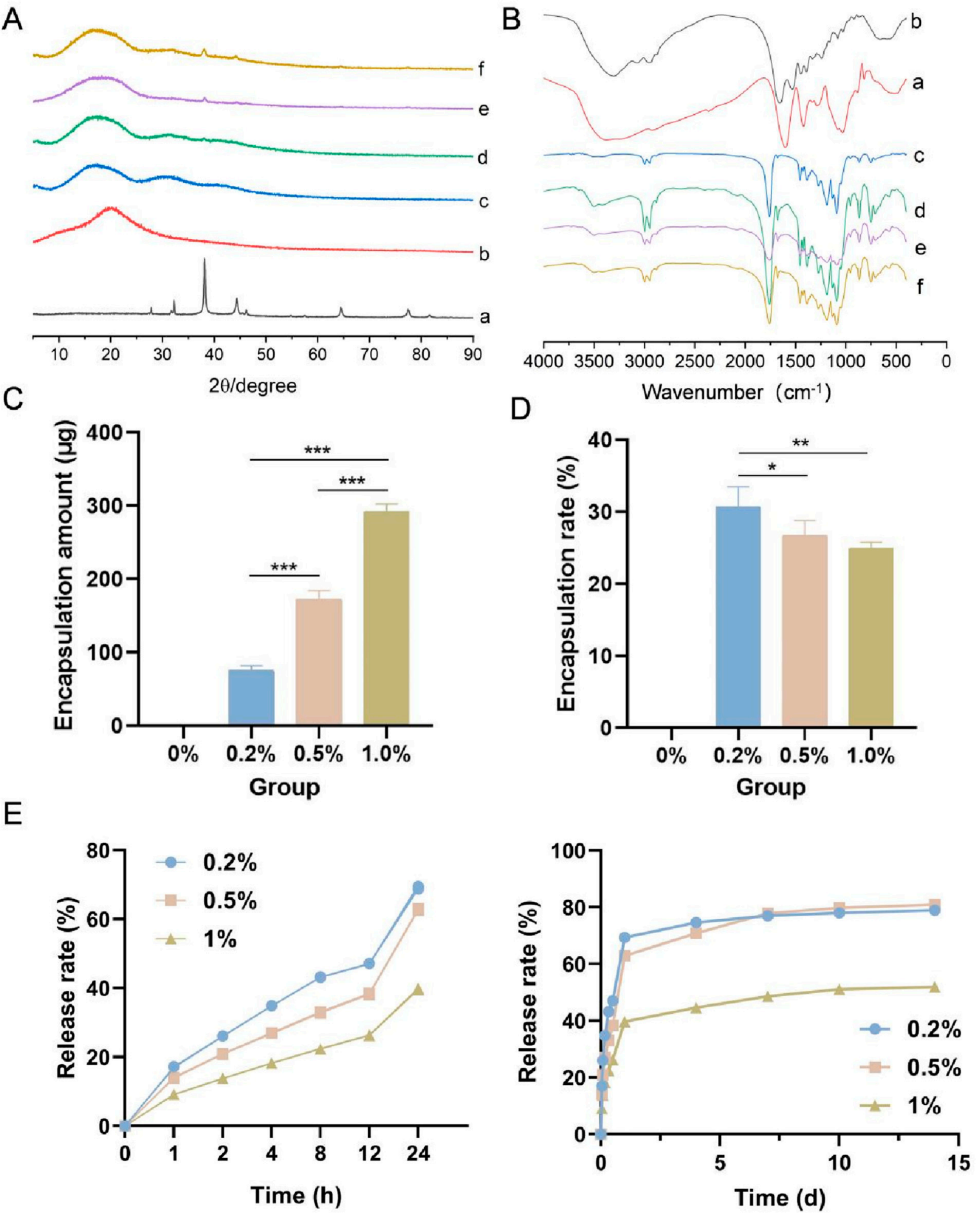
**(A)** X-ray diffraction patterns of the four types of membranes, the wool keratin powder and the nanosilver-loaded calcium alginate microsphere powder. **(B)** Fourier transform infrared spectra of the samples. **(C)** Amount of nanosilver encapsulated. **(D)** Nanosilver encapsulation rate. **(E)** Nanosilver release rate. a, nanosilver-loaded calcium alginate microsphere powder; b, wool keratin powder; c–f, 0%, 0.2%, 0.5% and 1.0% groups. * indicates *p* < 0.05, ** indicates *p* < 0.01, and *** indicates *p* < 0.001 (ANOVA).

The X-ray diffraction patterns of the four types of membranes, the wool keratin powder and the nanosilver-loaded calcium alginate microsphere powder are shown in Figure 2A. The characteristic diffraction peaks of the nanosilver-loaded calcium alginate microsphere powder were observed at 2θ = 38.15°, 44.32°, 64.47° and 77.53°, which are the (111), (200), (220) and (311) crystal plane diffraction peaks of face-centered cubic silver crystals, respectively (JCPDS, 04-0783) (Chen et al., 2022). The wool keratin powder, which was amorphous, displayed no obvious diffraction peaks. Among the four types of membranes, small diffraction peaks were observed at 2θ = 38.15° in the 0.2%, 0.5%, and 1.0% samples, and these peaks became more intense with increasing nanosilver-loaded calcium alginate microsphere content, whereas no diffraction peak was observed at 2θ = 38.15° in the 0% group. A small diffraction peak was also observed at 2θ = 44.32° in the 1.0% group. These results suggested that nanosilver was successfully incorporated into the electrospun membranes.

#### 3.1.3 FTIR spectroscopy

The FTIR spectra of the four types of membranes, the wool keratin powder and the nanosilver-loaded calcium alginate microsphere powder are shown in Figure 2B. Strong characteristic peaks for the wool keratin powder were observed at 3,306 cm^-1^ (N–H stretching vibration), and other characteristic peaks were observed at 1,658 cm^-1^ (C=O and C–N amide I stretching vibrations), 1,533 cm^-1^ (amide II N–H bending and C–N stretching vibrations) and 1,240 cm^-1^ (amide III C–N vibration and C=O bending vibrations) (Maurizii et al., 2024). A wide absorption peak was observed at approximately 3,374 cm^-1^ in the spectrum of the nanosilver-loaded calcium alginate microsphere powder, which was attributed to the stretching vibration caused by the intramolecular and intermolecular hydrogen bonds of the hydroxyl group (-OH). This peak also appeared in the spectra of the 0.2%, 0.5% and 1.0% groups, indicating the formation of hydrogen bonds between the hydroxyl groups. The four types of membranes also displayed peaks at 1758 cm^-1^, 1,186 cm^-1^, 1,089 cm^-1^, 2,997 cm^-1^ and 1,455 cm^-1^, which are the characteristic C=O, C-O-C, CH_3_ and CH_2_ peaks of PLGA, respectively (Wang et al., 2019). Because only a small amount of wool keratin powder was added to the four types of membranes, the characteristic peak of wool keratin powder was not observed in the spectra of the membranes.

#### 3.1.4 Amount of drug encapsulated and the drug encapsulation rate

The amount of nanosilver encapsulated and the nanosilver encapsulation rate in the four types of membranes are shown in Figures 2C,D. With increasing nanosilver content, the amount of nanosilver wrapped in the membrane gradually increased; the amount of nanosilver in the 1.0% group was the highest (92.55 ± 10.13 μg), whereas the amount of nanosilver in the 0.2% group was the lowest (75.41 ± 6.91 μg). With increasing nanosilver content, the encapsulation rate of nanosilver in the membrane decreased gradually, with the 0.2% group having the highest encapsulation rate.

#### 3.1.5 *In Vitro* analysis of nanosilver release

As shown in Figure 2E, the release of nanosilver from all three groups persisted for more than 14 days. Within the first 24 h, the release curves of all groups exhibited a rapid increase, presumably due to the nanosilver localized at or near the surface of the composite membranes. These nanosilver particles rapidly dissolved and diffused into the release medium, resulting in an initial burst release. In addition, water uptake caused the membranes to swell, increasing their volume and porosity, which provided more release channels for the nanosilver particles and thereby accelerated their release rate. Between days 2 and 14, the release curves gradually plateaued, indicating a decline in release rates, possibly due to the diminishing effects of diffusion and swelling. By day 14, the cumulative release rates for the 0.2%, 0.5%, and 1% groups were 78.92% ± 0.49%, 80.98% ± 0.29%, and 51.96% ± 0.20%, respectively. Notably, the 1% group exhibited a significantly slower release rate, which may be attributed to its higher nanosilver concentration, leading to reduced dispersibility and the formation of larger aggregates. These aggregates may form a saturated layer on the membrane surface, hindering further diffusion of nanosilver from the interior of the membrane to the surface and thus slowing the release rate.

#### 3.1.6 *In Vitro* analysis of antibacterial activity

**FIGURE 3:**
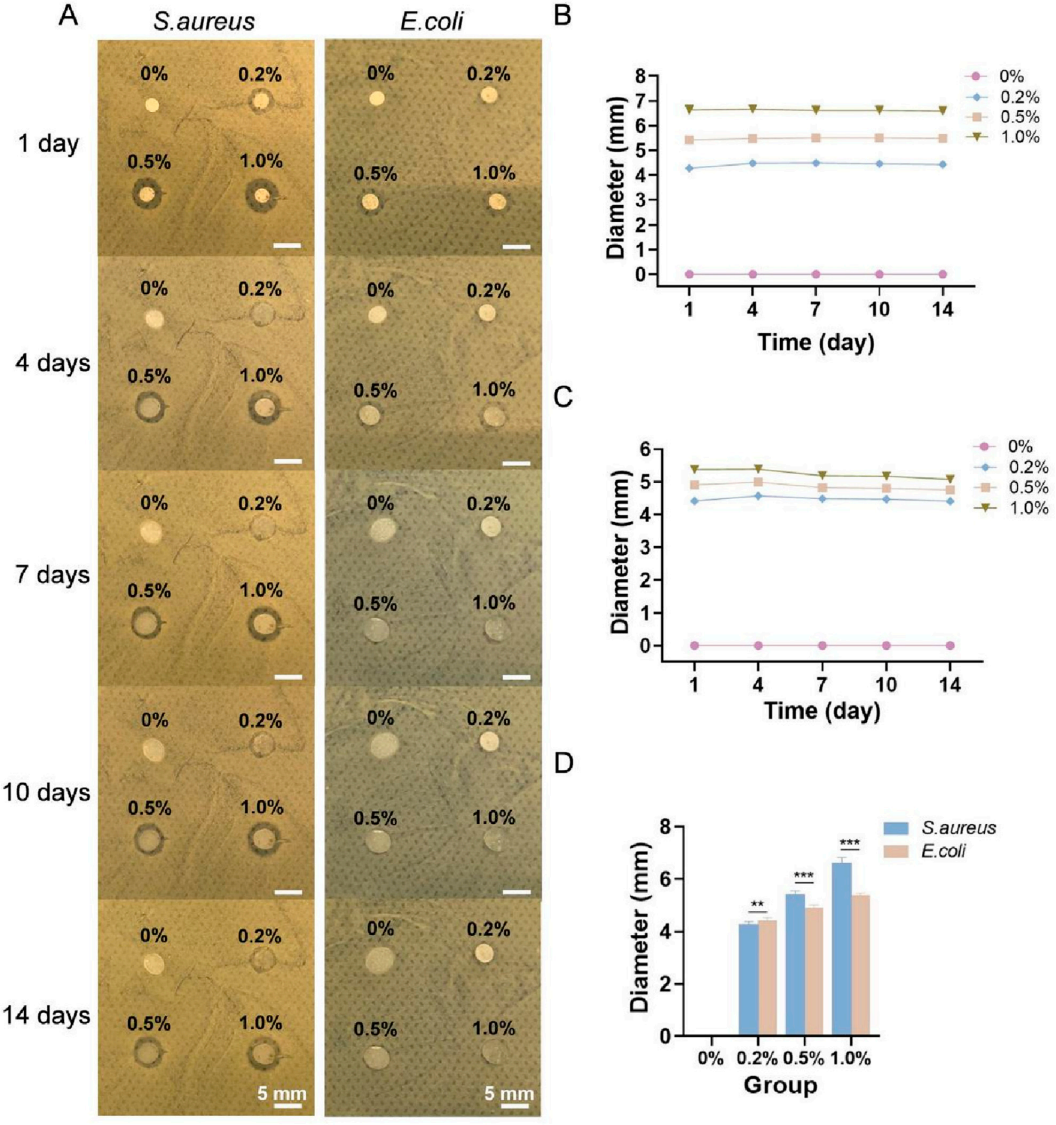
**(A)** General observations of the four types of membranes cocultured with *S. aureus* and *E. coli* for 1, 4, 7, 10 and 14 days. Diameters of the inhibition zones of each of the four types of membranes against *S. aureus* **(B)** and *E. coli* **(C)**. **(D)** Diameters of the inhibition zones of each type of membrane against *S. aureus* and *E. coli* on day 1. ** indicates *p* < 0.01, and *** indicates *p* < 0.001 (ANOVA).

The general observations of the four types of membranes cocultured with *S. aureus* and *E. coli* for 1, 4, 7, 10 and 14 days are shown in Figure 3A, and the diameters of the inhibition zones are shown in Figures 3B,C. No antibacterial zone was observed around the sample without nanosilver (0% group), whereas antibacterial zones appeared in the 0.2%, 0.5% and 1.0% groups, indicating that these membranes, but not the 0% membrane, inhibited the growth of *S. aureus* and *E. coli*. As the nanosilver content in the membrane increased, the diameter of the antibacterial zone also increased. The diameters of the inhibition zones for each of the four types of membranes against *S. aureus* and *E. coli* after 1 day are shown in Figure 3D. Among them, the 0.2% group presented stronger antibacterial activity against *E. coli* than *S. aureus*, whereas the opposite trend was observed in the 0.5% and 1.0% groups.

#### 3.1.7 Cytotoxicity

**FIGURE 4:**
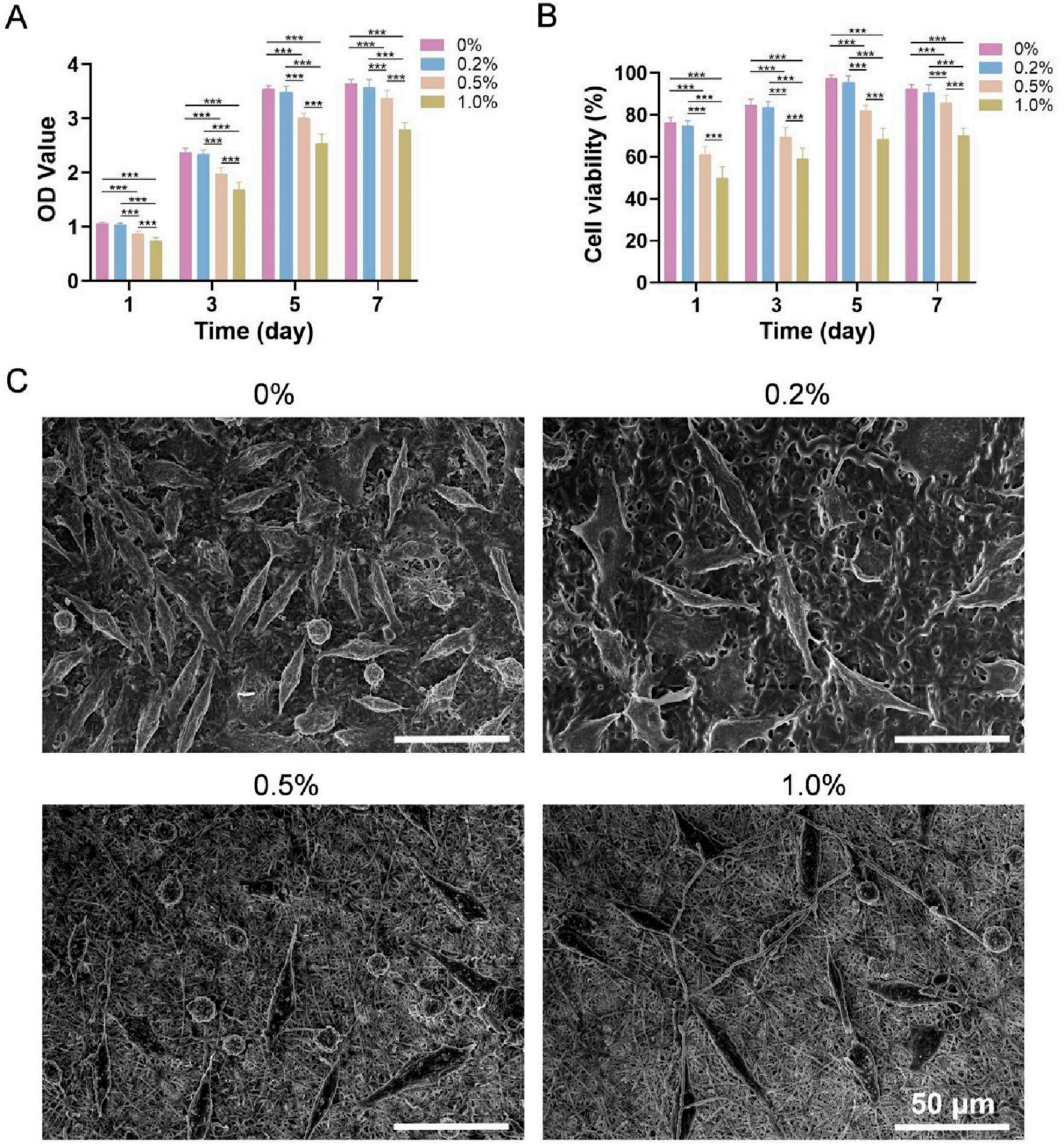
**(A)** OD values of each group after coculture with L-929 cells. **(B)** Cell viability. **(C)** SEM images of L-929 cells cultured on the four types of membranes for 3 days *** indicates *p* < 0.001 (ANOVA).

The OD values of the four types of membranes cocultured with L-929 cells are shown in Figure 4A. The OD value in each group increased with increasing culture duration, indicating that all four types of membranes promoted the adhesion and proliferation of L-929 cells. After 7 days, the 0% group and 0.2% group presented the greatest number of cells, followed by the 0.5% group and then the 1.0% group. The viability of the cells on the four types of membranes is shown in Figure 4B. Both the 0% and 0.2% groups were assigned Grade I cytotoxicity (mildly cytotoxic) after 1 day and Grade 0 (noncytotoxic) after 3, 5 and 7 days. Additionally, the 0.5% group was assigned Grade I after 1 and 3 days and Grade 0 after 5 and 7 days, whereas the cytotoxicity rating of the 1.0% group was determined to be Grade II (slightly cytotoxic) after 1 and 3 days and Grade I after 5 and 7 days.

#### 3.1.8 Cell adhesion

SEM images of L-929 cells cultured on the four types of membranes for 3 days are shown in Figure 4C. Most cells were elongated spindle-shaped or polygonal, and cell numbers were highest in the 0% and 0.2% groups, intermediate in the 0.5% group, and lowest in the 1.0% group, which is consistent with the CCK-8 results.

In summary, the four types of PLGA/WK electrospun membranes containing bFGF and different amounts of nanosilver were successfully prepared by emulsion electrospinning. The 0.2% group had the best drug encapsulation rate and release rate, sustained antibacterial activity and best cytocompatibility. Therefore, this membrane was selected to prepare a multifunctional bilayer dressing loaded with nanosilver and bFGF.

### 3.2 Characterization of the chitosan sponge containing bFGF

#### 3.2.1 Morphological observations

**FIGURE 5:**
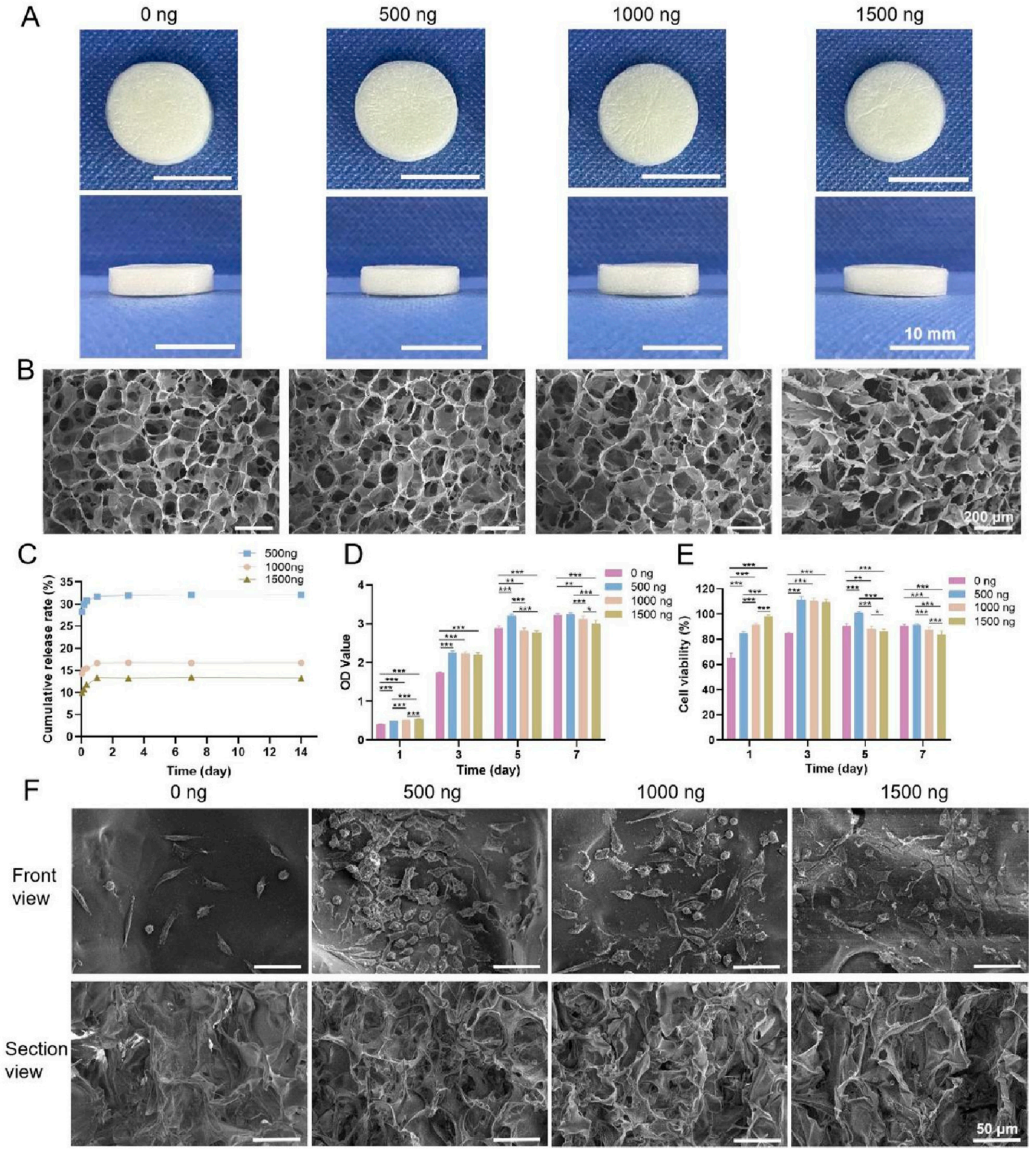
**(A)** Photographs of the four types of chitosan sponges. **(B)** Cross-sectional SEM images. **(C)** Cumulative release rates of bFGF. **(D)** OD values of the four types of chitosan sponges cocultured with L-929 cells. **(E)** Cell viability. **(F)** SEM images of L-929 cells cultured on the four types of sponges for 2 days * indicates *p* < 0.05, ** indicates *p* < 0.01, and *** indicates *p* < 0.001 (ANOVA).

Photographs of the four types of chitosan sponges are shown in Figure 5A. All of the sponges appeared milky white and spongy with dense structures and uniform textures and were approximately 1.3 cm in diameter and 0.3 cm in height. Cross-sectional SEM images of the four types of sponges are shown in Figure 5B. All the samples exhibited a porous network structure with pores that were uniform in size and interconnected. The pore sizes of the 0 ng, 500 ng, 1,000 ng and 1,500 ng groups were 47.46 μm, 49.57 μm, 46.34 μm and 43.02 μm, respectively, and their porosities were all greater than 90%, with values of 97.90%, 94.86%, 97.42% and 96.30%, respectively.

#### 3.2.2 *In Vitro* release of bFGF

The cumulative release rates of bFGF in the 500 ng, 1,000 ng and 1,500 ng groups are shown in Figure 5C (because the 0 ng group did not contain bFGF, this group was not included in this experiment). bFGF release can be divided into three main stages. In the first stage (8 h), bFGF displayed burst release, with cumulative release rates of 30.56% ± 0.28%, 15.65% ± 0.13% and 11.72% ± 0.11% in the 500 ng, 1,000 ng and 1,500 ng groups, respectively. The second stage occurred between 8 h and 24 h, when bFGF release was slow and sustained. During the third stage, after 24 h, bFGF release was stable, and the cumulative release rates in the 500 ng, 1,000 ng and 1,500 ng groups were 31.91% ± 0.27%, 16.78% ± 0.09% and 13.39% ± 0.13%, respectively. Thus, the cumulative release rate of bFGF in the 500 ng group was the highest, followed by that in the 1,000 ng group, while the 1,500 ng group presented the lowest cumulative release rate.

#### 3.2.3 Cytotoxicity

The OD values of the four types of chitosan sponges cocultured with L-929 cells are shown in Figure 5D. The OD values in each group increased with increasing culture duration, indicating that each of the sponges promoted L-929 cell adhesion and proliferation. After 1 day of coculture, the OD values increased, and the group with the highest bFGF content also had the highest OD value. The number of cells in the 1,500 ng group was the greatest, whereas the number of cells in the 0 ng group was the lowest. After 3 days, the numbers of cells in the 500 ng, 1,000 ng and 1,500 ng groups were greater than the number of cells in the 0 ng group, and after 5 and 7 days of coculture, the 500 ng group had the most cells. These results indicated that a moderate amount of bFGF promoted cell adhesion and proliferation, but excess bFGF inhibited cell adhesion and proliferation. The viability of the cells cultured on the four types of chitosan sponges is shown in Figure 5E. The cytotoxicity rating of the 0 ng group was Grade I after 1 day and Grade 0 after 3, 5 and 7 days; moreover, the cytotoxicity ratings of the 500 ng, 1,000 ng and 1,500 ng groups were Grade 0 after 1, 3, 5 and 7 days, indicating that these materials were noncytotoxic.

#### 3.2.4 Cell adhesion

SEM images of L-929 cells cultured on the four types of chitosan sponges for 2 days are shown in Figure 5F. The morphologies of the cells on the sponges were similar among the groups. Additionally, both cell number and density in the 500 ng, 1,000 ng, and 1,500 ng groups exceeded those in the 0 ng group, with the 500 ng group showing the highest and densest population.

In summary, four types of chitosan sponges containing bFGF were successfully prepared by vacuum freeze-drying. Among them, the 500 ng sponge displayed the best cumulative drug release rate and cytocompatibility. Therefore, this sponge was selected to prepare a multifunctional bilayer dressing coloaded with nanosilver and bFGF.

### 3.3 Characterization of the multifunctional bilayer dressing coloaded with nanosilver and bFGF

#### 3.3.1 Morphological observations

**FIGURE 6:**
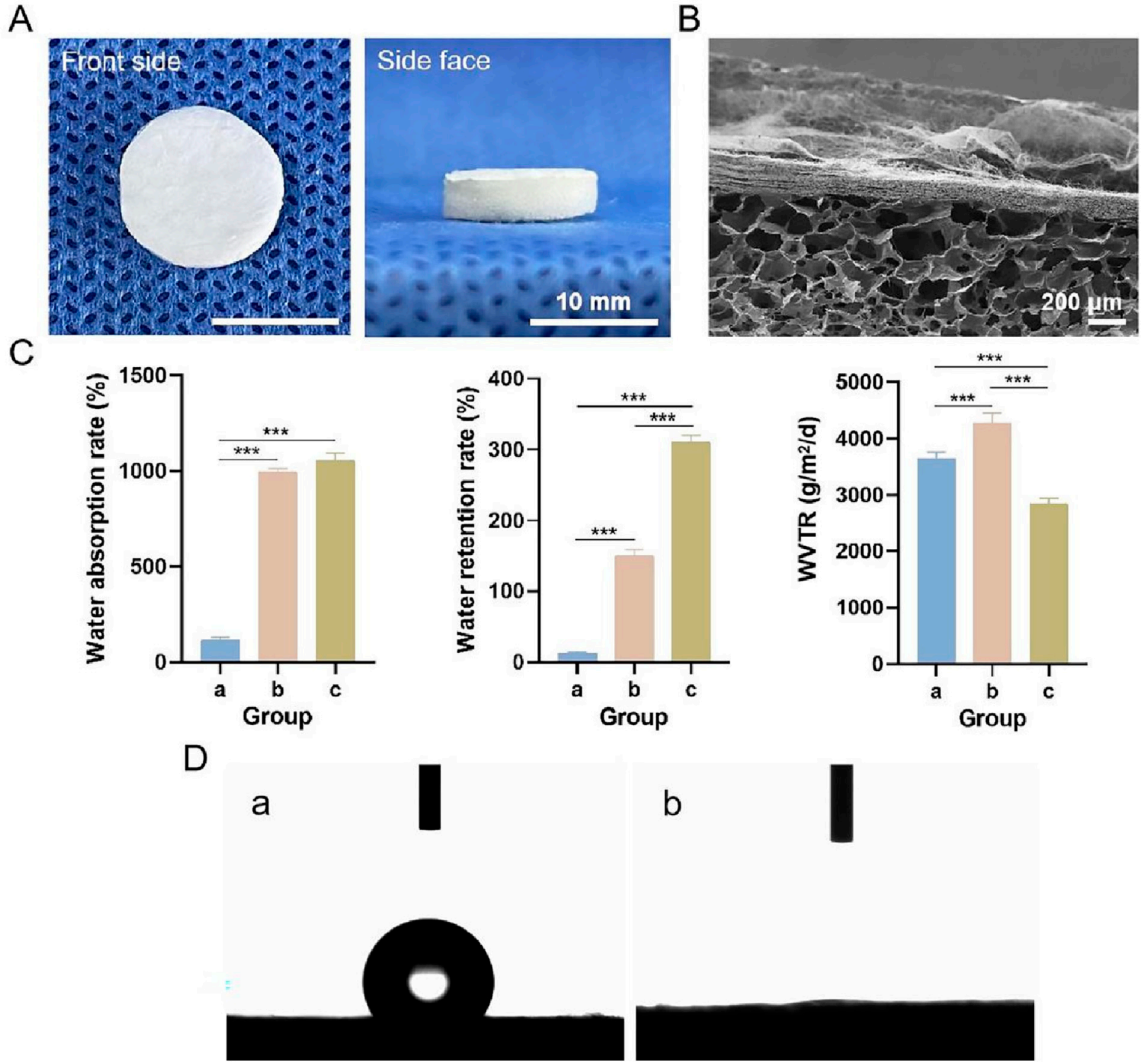
**(A)** Photographs of the multifunctional bilayer dressing coloaded with nanosilver and bFGF. **(B)** Cross-sectional SEM image. **(C)** Water absorption rate, water retention rate and WVTR. **(D)** Images from the water contact angle assessment. a–c indicate the upper layer, lower layer and bilayer dressing, respectively. *** indicates *p* < 0.001 (ANOVA).

Photographs of the bilayer dressing, which was approximately 1.3 cm in diameter and 0.3 cm in height, are shown in Figure 6A. The upper layer was composed of a PLGA/WK electrospun membrane containing 0.2% nanosilver and bFGF, and the lower layer was composed of a chitosan sponge containing 500 ng of bFGF. The cross-sectional SEM image of the bilayer dressing is shown in Figure 6B. The upper and lower layers were close together and not easily separated.

#### 3.3.2 Water absorption rate, water retention rate and WVTR

The water absorption rate, water retention rate and WVTR of each sample are shown in Figure 6C. The water absorption rates of the bilayer dressing and lower layer were high, whereas the water absorption rate of the upper layer was low. This difference is because the bilayer dressing and lower layer have loose, spongy structures with a strong water absorption capability. Additionally, the water retention rate of the bilayer dressing was the highest, and the water retention rate of the upper layer was the lowest, whereas the WVTR of the lower layer was the highest and that of the bilayer dressing was the lowest.

#### 3.3.3 Water contact angles

Images from the bilayer dressing water contact angle evaluation are shown in Figure 6D. The water contact angle of the upper layer was 121.34° ± 2.58°, indicating its hydrophobicity, which can simulate the epidermis of the skin and prevent bacterial invasion. The lower layer was hydrophilic with a water contact angle of 0°, and this layer can simulate the dermis of the skin and better absorb exudate.

#### 3.3.4 Bacterial penetration

**FIGURE 7:**
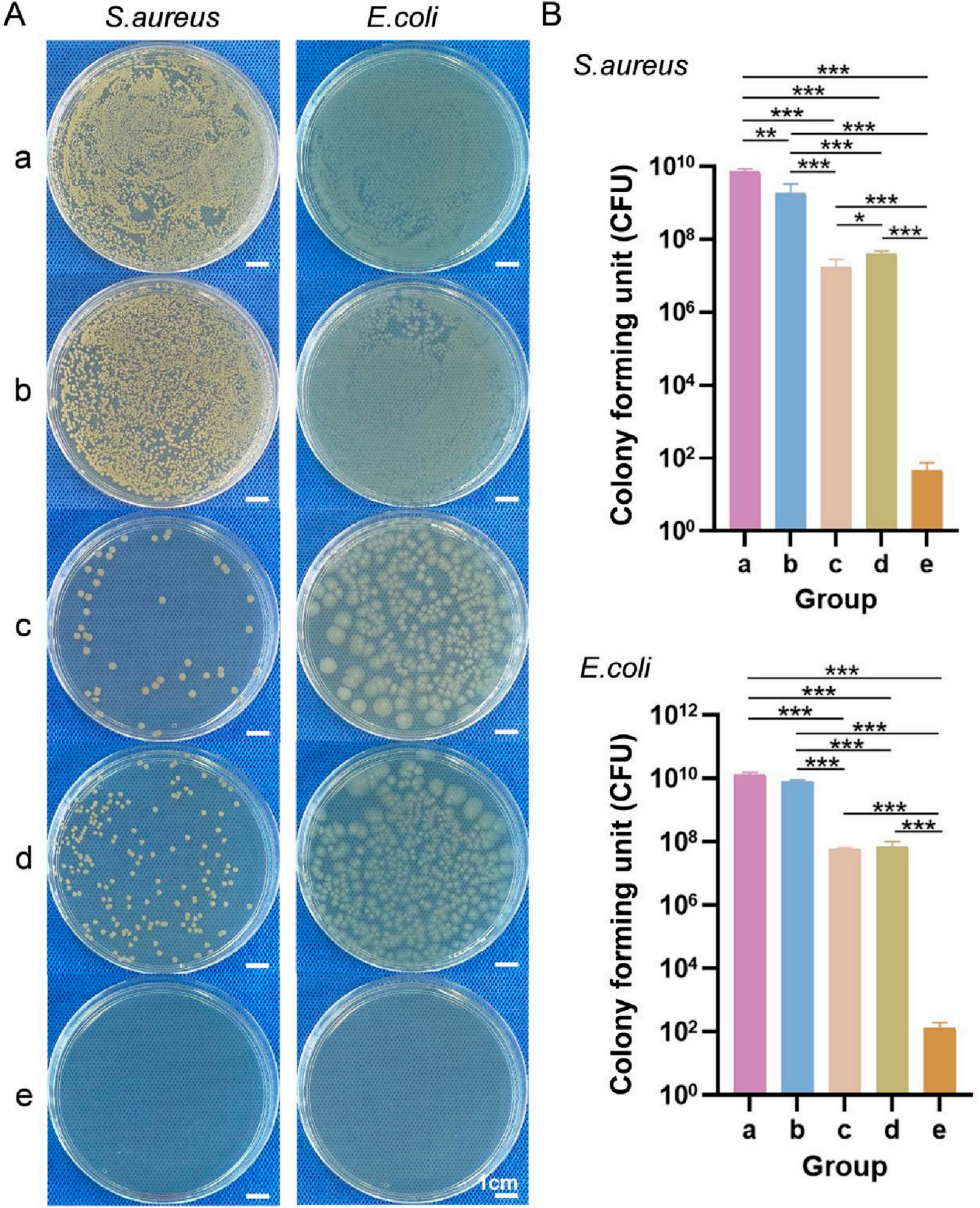
**(A)** General observations from the *S. aureus* and *E. coli* penetration tests. **(B)** Numbers of *S. aureus* and *E. coli* colonies that penetrated each of the samples. a–e are the blank control, sterile gauze, upper layer, lower layer, and bilayer dressing, respectively. * indicates *p* < 0.05, ** indicates *p* < 0.01, and *** indicates *p* < 0.001 (ANOVA).

General observations from the *S. aureus* and *E. coli* penetration tests are shown in Figure 7A. Moreover, the numbers of *S. aureus* and *E. coli* colonies that penetrated each sample are shown in Figure 7B. Fewer *S. aureus* and *E. coli* colonies penetrated the upper layer, lower layer, and bilayer dressing than the blank control and sterile gauze. Except for the blank control group, the number of *S. aureus* colonies that penetrated the bilayer dressing was the lowest, with an average of 80 CFUs, followed by the upper layer and then the lower layer. The sterile gauze allowed most of the *S. aureus* colonies to penetrate, with an average of 7.07 × 10^9^ CFUs. Similarly, for *E. coli*, when the blank control group was excluded, the number of colonies that penetrated the bilayer dressing was the lowest, and the number of colonies that penetrated the sterile gauze was the greatest. The results indicated that the bilayer dressing had an excellent ability to prevent bacterial penetration.

#### 3.3.5 Cell scratch wound healing test

**FIGURE 8:**
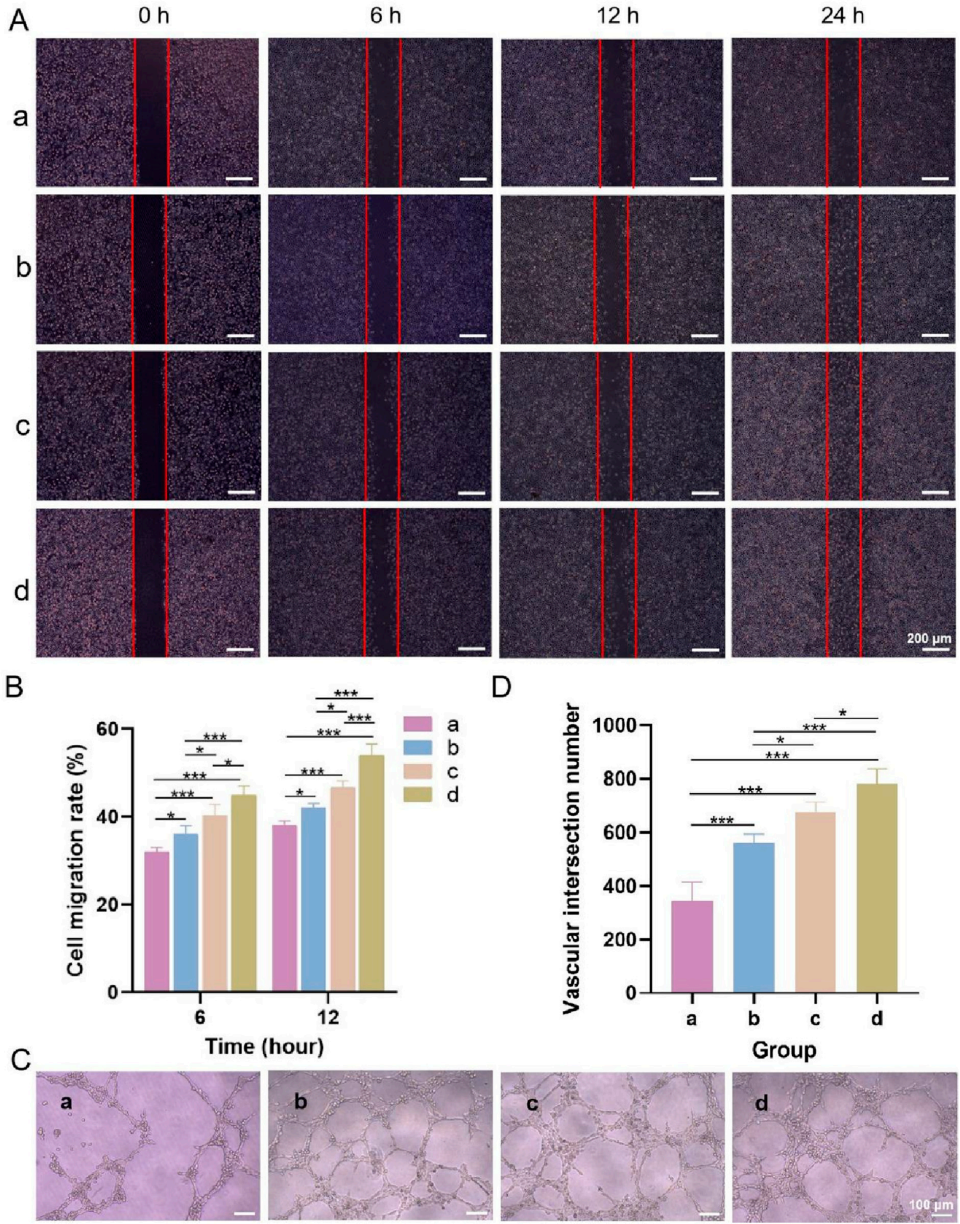
**(A)** Microscopic observations of cell scratch wound healing. **(B)** Cell migration rates. **(C)** Microscopic observations of angiogenesis. **(D)** Number of vascular junctions. a–d are the blank control, upper layer, lower layer, and bilayer dressing, respectively. * indicates *p* < 0.05, and *** indicates *p* < 0.001 (ANOVA).

Figure 8A shows microscopy images of the cell scratch wound healing, and Figure 8B shows the cell migration rates of each group. Compared with that in the blank control group, fibroblast migration was accelerated in the other three groups. After 6 and 12 h, the cells in the upper layer, lower layer and bilayer dressing groups tended to migrate to the scratch area. Notably, the cells in the bilayer dressing group migrated the fastest, followed by those in the lower layer group, and the cells in the blank control group migrated the slowest. After 24 h, the scratches in the lower layer and bilayer dressing groups had essentially healed.

#### 3.3.6 Angiogenesis test

Microscopy observations of angiogenesis and the number of vascular junctions in each group are shown in Figures 8C,D. Tubular structures formed in the three experimental groups but not in the blank control group. Among them, the bilayer dressing group had the greatest number of vascular junctions, followed by the lower layer group and then the upper layer group.

#### 3.3.7 *In Vitro* coagulation experiment

**FIGURE 9:**
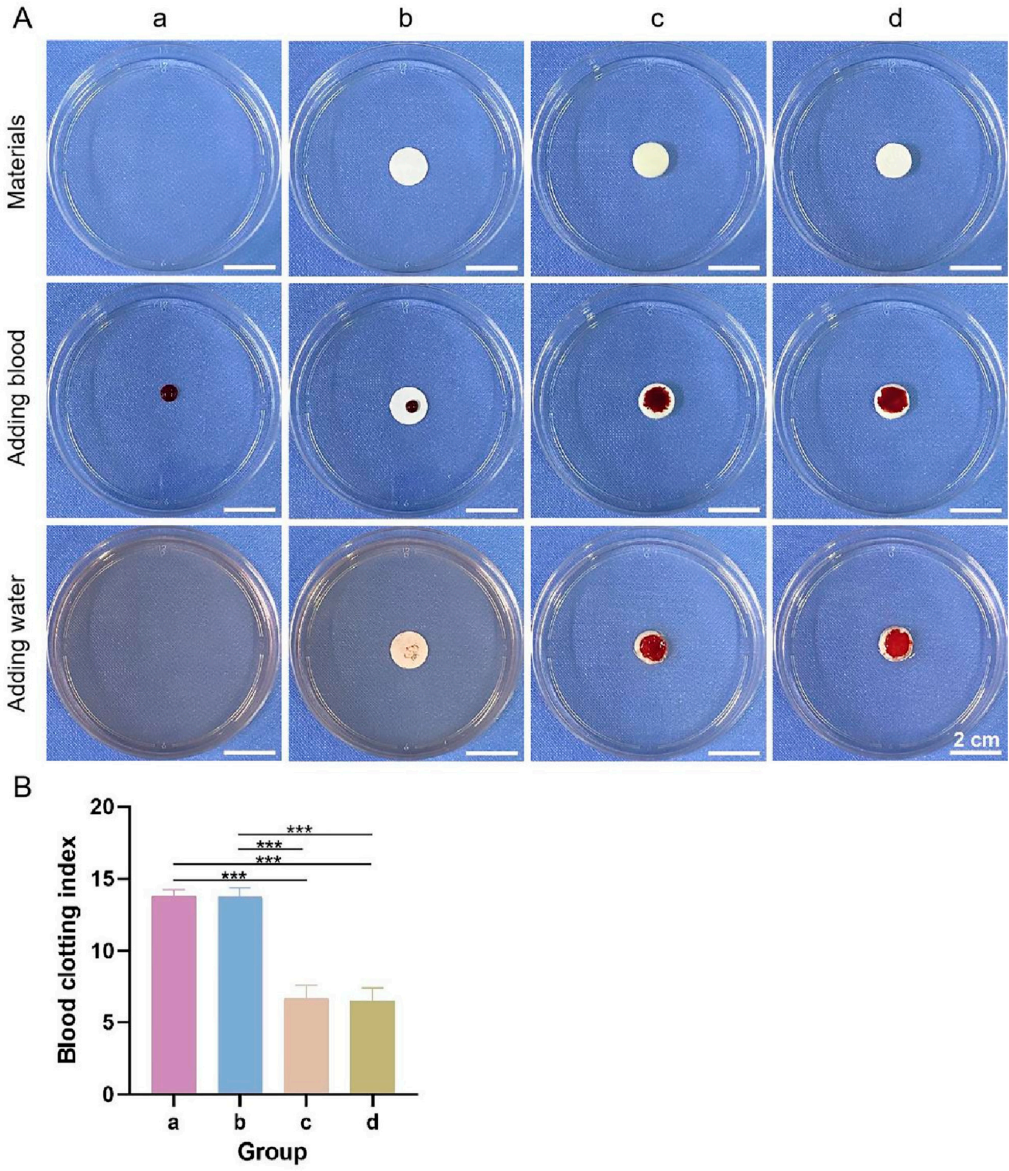
**(A)** General observations from the coagulation test. **(B)** Blood clotting indices. a–d are the blank control, upper layer, lower layer, and bilayer dressing, respectively. *** indicates *p* < 0.001 (ANOVA).

General observations from the coagulation test are shown in Figure 9A, and the blood clotting index of each group is shown in Figure 9B. The color of the lower layer and the bilayer dressing groups did not change significantly after the addition of deionized water, whereas the liquids in the other two groups were obviously pink, indicating that the former two groups had better coagulation performance. In addition, the blood clotting indices of the former two groups were significantly lower than those of the latter two groups. The lower the blood clotting index is, the better the coagulation performance. These results showed that the lower layer and bilayer dressing had good coagulation properties.

### 3.4 Repair of full-tthickness skin wounds

#### 3.4.1 Gross observations and wound healing rate

**FIGURE 10:**
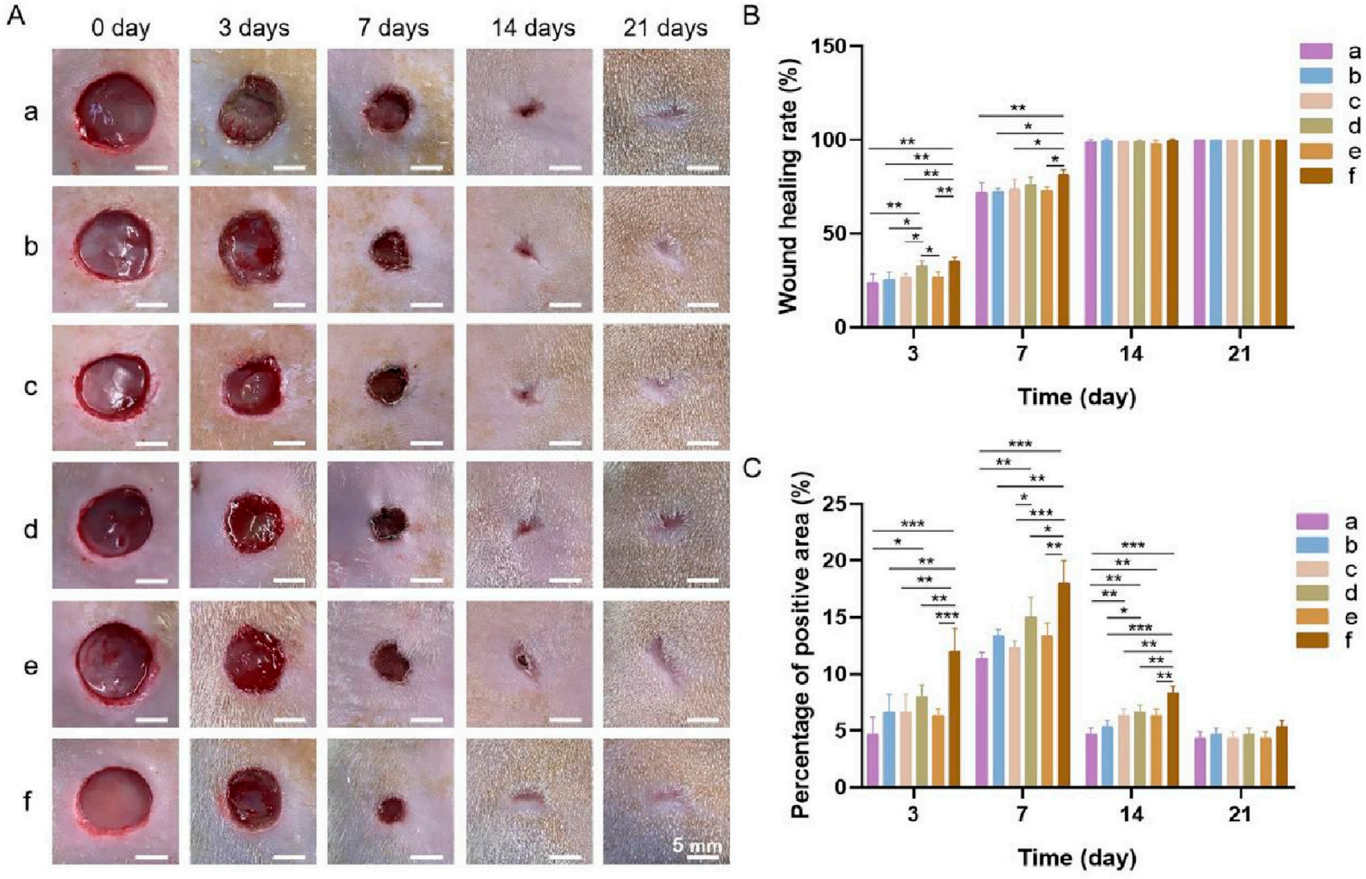
**(A)** General observations during wound healing. **(B)** Wound healing rates. **(C)** Percentage of the area positive for PCNA. a, blank control; b, bilayer dressing without drugs; c, bilayer dressing coloaded with nanosilver and bFGF in the upper layer; d, bilayer dressing loaded with bFGF in both the upper layer and lower layer; e, bilayer dressing loaded with bFGF in the upper layer; f, bilayer dressing coloaded with nanosilver and bFGF. * indicates *p* < 0.05, ** indicates *p* < 0.01, and *** indicates *p* < 0.001 (ANOVA).

The gross observations during wound healing and the wound healing rates in each group at different time points are shown in Figures 10A,B. The wound healing rate of each group increased with time. After 3 days, the wound healing rates were the highest in groups d and f at 32.64% ± 2.83% and 35.28% ± 2.06%, respectively. After 7 days, group f displayed the highest wound healing rate. By day 14, the wounds in all the groups had essentially healed, and a significant difference in the wound healing rate was not observed among the groups. After 21 days, the wounds in all the groups had completely healed. The results showed that the bilayer dressing coloaded with nanosilver and bFGF (group f) synergistically accelerated wound healing.

#### 3.4.2 Histological examination

**FIGURE 11:**
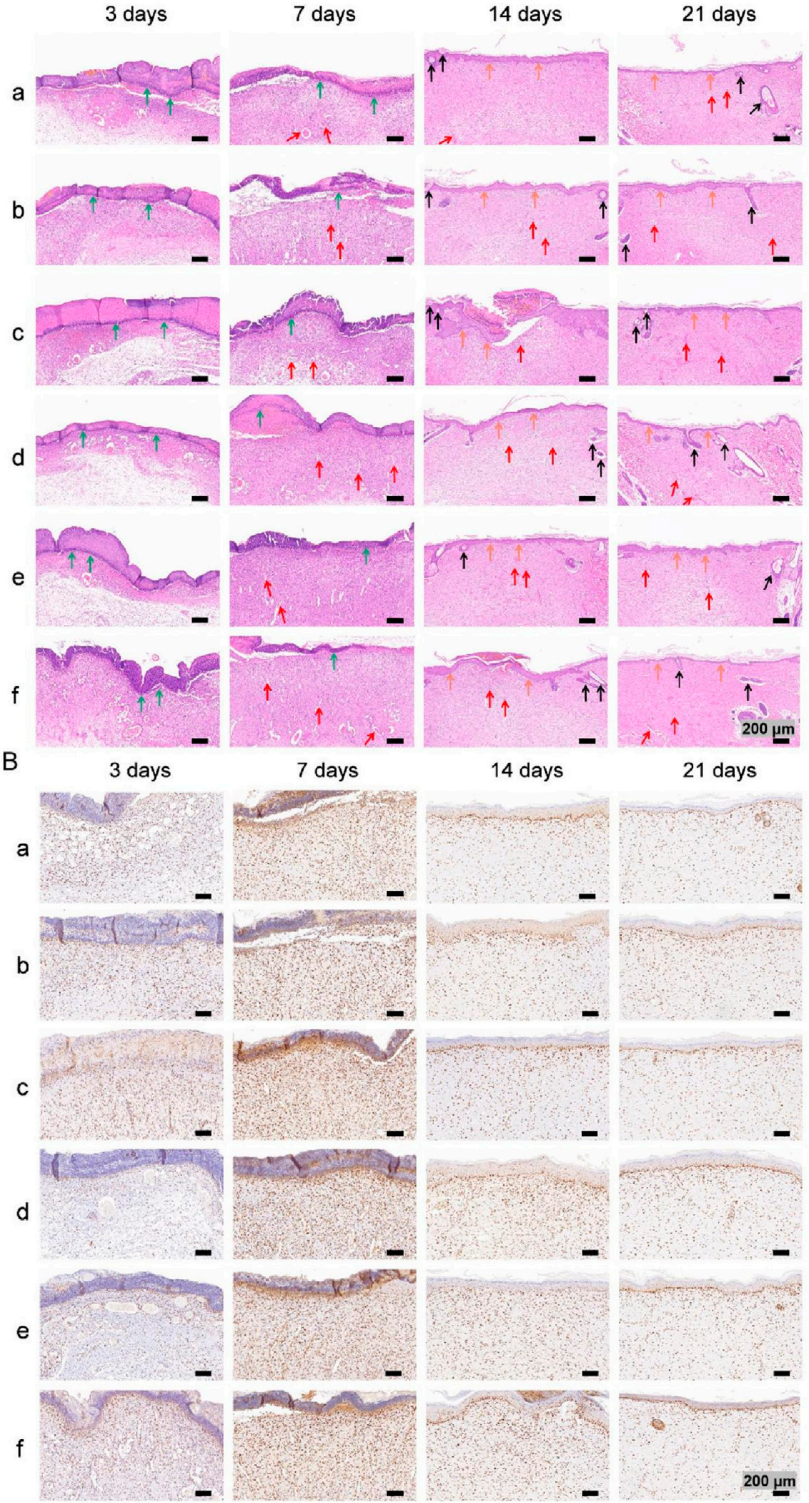
**(A)** Images of HE staining. The green, red, black and orange arrows denote inflammatory cells, new blood vessels, hair follicles and new epidermal tissue, respectively. **(B)** Images of PCNA immunohistochemical staining images.

Images of HE-stained sections from each group are shown in Figure 11A. Inflammatory cell infiltration was observed in all the groups after 3 days. At 7 days, fibroblasts and many new capillaries were observed in groups d and f, along with significantly fewer inflammatory cells, whereas fewer new capillaries were observed in the other groups. At 14 days, new hair follicles were observed in groups d and f, but fewer hair follicles were observed in the other groups. After 21 days, the structure of the epidermis in each group was continuous and complete and similar to that of normal skin, and skin appendages, such as hair follicles, were also observed in all the groups, indicating that wound repair was essentially complete.

Images of immunohistochemical staining for PCNA and the percentages of the area positively stained for PCNA in each group are displayed in Figures 11B, 10C. PCNA is one of the main markers of cell proliferation and plays a key role in the initiation of cell proliferation; thus, PCNA expression reflects the conditions of cell proliferation (Hao et al., 2020). Compared with that in the other groups, PCNA expression in each group increased with time over 7 days and was significantly higher at 3 and 7 days in group f. These findings indicate that the application of both nanosilver and bFGF promoted the maintenance of cell proliferation and effectively promoted fibroblast proliferation and migration in the early stage of wound healing. On days 14 and 21, the expression of PCNA in each group decreased, indicating that in the late stage of wound healing, the tissue had gradually been remodeled because of collagen deposition and extracellular matrix formation, and the demand for fibroblasts decreased.

#### 3.4.3 Western blot

**FIGURE 12:**
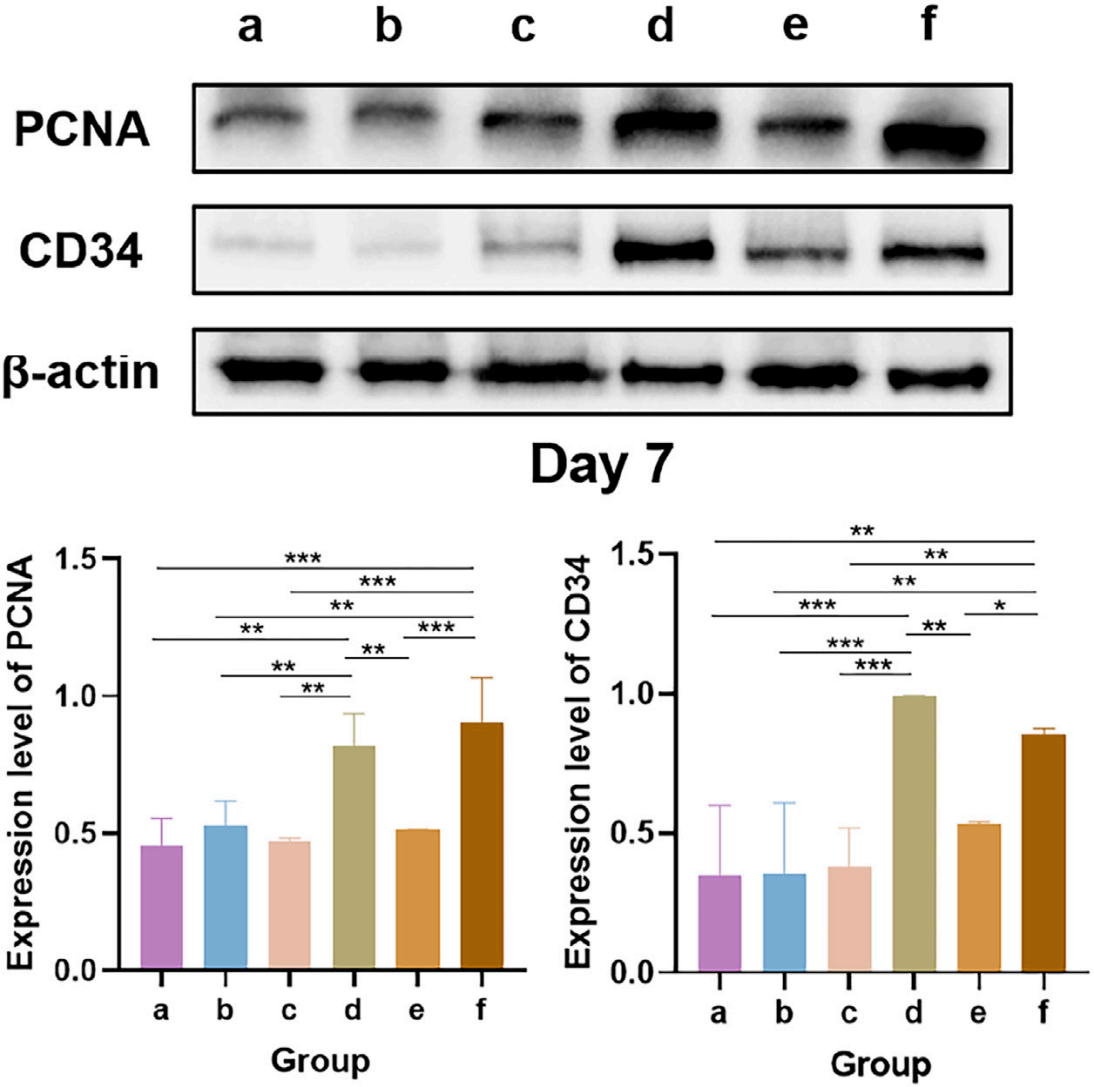
Western blot analysis of PCNA and CD34 levels. * indicates *p* < 0.05, ** indicates *p* < 0.01, and *** indicates *p* < 0.001 (ANOVA).

The results of the Western blot analysis of PCNA and CD34 levels at 7 days are shown in Figure 12. PCNA is an indicator for assessing the proliferative status of cells, and its expression level objectively reflects cellular proliferative activity. CD34, a well-established marker of vascular endothelial cells, labels endothelial cells and immature small vessels, thereby reflecting the extent of angiogenesis (Zhang et al., 2022). Western blot analysis revealed that the bFGF-loaded wound dressings (groups d and f) significantly upregulated the expression of both PCNA and CD34. These findings are consistent with those of several previous studies: high PCNA expression reflects increased cellular proliferative activity, which can be attributed to the direct stimulation of growth factor signaling pathways, such as collagen synthesis and epidermal regeneration, by bFGF, thereby attenuating oxidative stress and increasing the levels of matrix metalloproteinases (MMPs) in the wound microenvironment to ultimately accelerate re-epithelialization (Yin et al., 2024). In addition, bFGF promotes the infiltration of CD34-positive cells, thereby enhancing angiogenesis and granulation tissue formation and accelerating wound closure (Oshima et al., 2024). Collectively, the concerted upregulation of PCNA and CD34 expression underscores the therapeutic potential of the bFGF-loaded dressings, which effectively shorten the wound-healing timeline by increasing angiogenesis and cellular proliferation.

## 4 Discussion

An ideal wound dressing should not only effectively absorb excess exudate but also maintain a moist environment, thus accelerating the healing process. In this study, compared with the upper layer group, the lower layer and the bilayer dressing groups had better water absorption performance. This difference occurred because the structures of the latter two groups were loose and spongy with many pores, which is conducive to absorbing liquids (He et al., 2021). The WVTR is also an important index for testing whether a material is suitable for use as a wound dressing (Varshney et al., 2022). The WVTRs of normal skin and damaged skin are 204 g/(m^2^·d) and 279–5,138 g/(m^2^·d), respectively, and the ideal WVTR of medical dressings is approximately 2,500 g/(m^2^·d) (M.B. Al-Sheerazee et al., 2024). The WVTR of the bilayer dressing prepared in this study was 2,854 g/(m^2^·d), which meets the requirements of wound dressings in terms of water vapor transmittance.

Interestingly, the 500 ng bFGF-loaded chitosan sponge exhibited the highest cumulative release rate (≈32%) at 24 h, whereas those of the 1,000 ng and 1,500 ng groups plateaued at only ≈17% and ≈13%, respectively (p < 0.001). This inverse relationship between the loading dose and fractional release may be rationalized by two synergistic phenomena described in previous mechanistic studies. First, the positively charged amine groups of chitosan establish a finite number of electrostatic/hydrogen-bonding sites for the anionic patches of bFGF (Masuoka et al., 2005); this interaction follows a Langmuir-type isotherm with a saturation threshold. Below this threshold, virtually all bFGF molecules are individually immobilized and remain readily accessible to the release medium (Shajahan et al., 2017). Once the dose exceeds the saturation point, the excess protein exists primarily as free solute that can self-associate during the second freeze-drying step (Wang et al., 2010). Second, these supramolecular aggregates precipitate on the scaffold walls, markedly narrowing or occluding the pores of the sponge. The resulting increase in the effective diffusion path length and decrease in pore connectivity substantially attenuate further bFGF release (Johnson et al., 1996). Collectively, the balance between saturative adsorption and aggregation-induced pore blockage explains why loading a lower amount of bFGF (500 ng) yields the highest fractional release, whereas higher doses paradoxically suppress it. Furthermore, the kinetics of bFGF release can influence the trajectory of wound repair. The rapid but nonexhaustive burst (≈30% within 8 h) delivered by the 500 ng sponge provides an early surge of mitogen necessary for the initial recruitment of fibroblasts and endothelial cells during the inflammatory-to-proliferative transition (Kanda et al., 2014). Importantly, the subsequent sustained low-level release of bFGF establishes a “steady-state micro-depot” within the wound bed, ensuring continuous pro-angiogenic and pro-proliferative signaling while avoiding receptor downregulation and hypertrophic scarring associated with high-dose bolus administration. In contrast, the 1,500 ng sponge, despite its higher payload, delivers only ≈13% of the load by 24 h and may fail to trigger robust angiogenesis (Morimoto et al., 2013). Moreover, the large residual depot retained in the scaffold risks late, uncontrolled release once the sponge begins to degrade, potentially inducing hypergranulation or fibrosis (Tabata, 2009). Therefore, the superior early-to-mid phase release of the 500 ng formulation not only maximizes therapeutic efficiency but also minimizes the risk of bFGF overdose.

Most wound infections are caused by *S. aureus* (a gram-positive bacterium) and *E. coli* (a gram-negative bacterium). Enhancing the antibacterial properties of a wound dressing can effectively increase its clinical application value (Lu et al., 2022). The multifunctional bilayer dressing prepared in this study effectively prevented the penetration of *S. aureus* and *E. coli*. In addition, the dressing showed good antibacterial activity against these two bacterial species.

Fibroblast migration and proliferation are crucial during wound healing (Robinson et al., 2014). The results of the cell scratch wound healing assay revealed that the bilayer dressing significantly accelerated fibroblast migration, mainly because the bFGF released from the dressing activated fibroblast proliferation and migration through various signaling pathways. bFGF can activate the Hedgehog signaling pathway by regulating the Smoothened (Smo) receptor, which acts upstream of phosphoinositol 3-kinase (PI3K)–c-Jun N-terminal kinase (JNK)–β-catenin signaling to promote the migration of fibroblasts (Zhu et al., 2017). Furthermore, bFGF can accelerate fibroblast migration by activating the Wnt/β-catenin signaling pathway (Wang et al., 2017).

The whole-blood coagulation index can be used to evaluate whether materials have hemostatic properties (Hao et al., 2020). Chitosan is among the most widely used hemostatic polysaccharides. Owing to its unique molecular structure with free amino groups, chitosan is positively charged and can bind negatively charged red blood cells, resulting in red blood cell aggregation and rapid hemostasis (Chen et al., 2020). The hemostatic performance of the bilayer dressing was good because its lower layer was composed of a chitosan sponge.

Wound healing encompasses a series of very complex dynamic processes, of which the wound healing rate and degree of epidermal re-epithelialization are important indicators of the extent of wound repair (Hou et al., 2022). During the process of wound closure, some fibroblasts gradually proliferate and differentiate into myofibroblasts, contact the matrix at the edge of the wound and generate contraction forces, which pull the edge of the wound toward the center and gradually reduce the wound area (Fu et al., 2024). In addition, keratinocytes proliferate and migrate at the wound site to gradually cover the wound and form a new epidermal layer, which results in re-epithelialization of the wound surface (Wang et al., 2022). PCNA expression was the highest after 3 and 7 days in group f, and this group also had the highest wound healing rate after 7 days. Furthermore, a new epidermal layer with a continuous and complete structure similar to that of normal skin formed in group f after 21 days, indicating that the combination of bFGF and nanosilver maintained the good proliferative state of the cells to synergistically accelerate wound healing. On the one hand, bFGF can significantly promote the proliferation and differentiation of fibroblasts, endothelial cells and keratinocytes, as well as the expression and release of related factors (Bian et al., 2023). In addition, bFGF activates the migration of inflammatory cells to wounds by increasing the expression of different chemokines, which is helpful in the early stage of inflammation (Bian et al., 2023). On the other hand, nanosilver has antibacterial properties, especially during the early inflammatory period, and can effectively prevent infection while providing a good environment for wound healing. In addition, nanosilver can induce the proliferation and migration of keratinocytes and promote the differentiation of fibroblasts into myofibroblasts, thus contributing to wound contraction and closure (Kalaoglu-Altan et al., 2021). Moreover, nanosilver can upregulate the expression of the most important proinflammatory cytokines, namely, IL-1 (α and β), IL-6 and IL-8, to initiate the wound healing process (Kalaoglu-Altan et al., 2021).

Aquacel^®^ Ag Extra is a commercial antimicrobial hydrofiber dressing composed of a single layer of sodium carboxymethylcellulose (CMC) fibers impregnated with 1.2% ionic silver. Luze et al. used a porcine excisional wound model (3 × 3 cm deep, 1.2 mm deep) to analyze the effect of antiseptic-loaded bacterial nanocellulose on the healing process (Luze et al., 2022). Aquacel^®^ Ag Extra was used as a positive control, and the wound healing rate in this group reached approximately 75% after 7 days. In contrast, the bilayer dressing coloaded with nanosilver and bFGF prepared in this study was used for full-thickness skin defect (a full-thickness skin defect with a diameter of 1 cm) repair in rats, and the wound healing rate reached 81.52% ± 2.53% at 7 days, which was better than that of Aquacel^®^ Ag Extra, probably because bFGF and nanosilver maintained the good proliferative state of the cells to synergistically accelerate wound healing. However, the differences in the experimental animal models and wound specifications may be among the factors contributing to the differences in the healing rate.

## 5 Conclusion

In this study, a multifunctional bilayer dressing with an upper layer composed of a PLGA/WK electrospun membrane containing 0.2% nanosilver and bFGF and a lower layer composed of a chitosan sponge containing 500 ng of bFGF was successfully prepared by emulsion electrospinning and vacuum freeze-drying. The upper layer was hydrophobic, while the lower layer was hydrophilic. The bilayer dressing had a high water absorption rate, an appropriate water retention rate and a suitable WVTR. In addition, the dressing effectively prevented bacterial penetration, had good antibacterial activity, accelerated fibroblast migration, and promoted angiogenesis and coagulation. Moreover, the bilayer dressing coloaded with nanosilver and bFGF shortened the wound healing time and significantly promoted wound healing synergistically.

## Data Availability

The raw data supporting the conclusions of this article will be made available by the authors, without undue reservation.
